# Antibiotics in the Treatment of Periodontitis: A Systematic Review of the Literature

**DOI:** 10.1155/2021/6846074

**Published:** 2021-11-08

**Authors:** Chaima Hammami, Wafa Nasri

**Affiliations:** University of Monastir, Dental Medecine Faculty of Monastir, Periodontal Departement, Oral Health and Oral Rehabilitation Research Laboratory, LR12ES11, Monastir 5019, Tunisia

## Abstract

**Introduction:**

Systemic antibiotics present one of the alternative adjunctive therapies in nonsurgical periodontal treatment (NSPT). Different protocols have been proposed, but their indication and effectiveness are still controversial. The aim of this study is to assess the effectiveness of the addition of antibiotics after nonsurgical debridement during initial therapy and compare different antimicrobial prescription protocols.

**Materials and Methods:**

An electronic search was performed through MEDLINE and EBSCOhost databases using the appropriate MeSH words. The target studies have to be published during the last five years. Data from the selected studies were extracted and analyzed. Study selection was done based on inclusion and exclusion criteria.

**Results:**

Seven randomized clinical trials were included in our review. Their data were extracted using a grid established for this purpose. Collectively, different protocols have been proposed and almost all of them yield superior clinical and microbiological results compared to the placebo group.

**Conclusion:**

The overall findings of this review show a positive effect of the use of antibiotics as an adjunctive to NSPT, regardless of the antimicrobial agents used in our included studies. Sites with PD > 6 mm may benefit most from the adjunctive use of antibiotics in NSPT. This trial is registered with Clinicaltrials.gov identifiers: NCT02829983 (Bechara Andere et al., 2016); NCT02839421 (Ardila et al., 2020); NCT02735395 (Borges et al., 2017); NCT02359721 (Suryaprasanna et al., 2018); and NCT01318928 (Hans, 2015).

## 1. Introduction

Periodontitis is defined as an inflammatory disease of supporting tissues of the teeth caused by specific microorganisms or groups of specific microorganisms, resulting in progressive destruction of the periodontal ligament and alveolar bone with periodontal pocket formation, gingival recession, or both [1].

Periodontal diseases are polymicrobial, multifactorial diseases, and there are many host factors involved in determining the individual susceptibility to disease. It is recognized that the relationship between periodontal microbiota and the host is generally benign but, when the specific bacterial species overgrows in the subgingival spaces, this may cause periodontal inflammation and destruction with attachment loss and bone loss [2].

A destructive periodontal inflammation could occur because of the dysregulation of the immune fitness and, as a result further induction of microbial dysbiosis would be noticed. This latter heightens back the immune response asin a vicious cycle [3].

Recent advancements in the periodontal research field are consistent with a new model of pathogenesis according to which periodontitis is initiated by a synergistic and dysbiotic microbial community rather than by select “periopathogens,” such as the “red complex.” In this polymicrobial synergy, different members or specific gene combinations within the community fulfill distinct roles that converge to shape and stabilize a disease-provoking microbiota. One of the core requirements for a potentially pathogenic community to arise involves the capacity of certain species, termed “keystone pathogens,” to modulate the host response in ways that impair immune surveillance and tip the balance from homeostasis to dysbiosis. Keystone pathogens also elevate the virulence of the entire microbial community through interactive communication with accessory pathogens [4].

During the inflammatory response, IL-6 is one of the main host inflammatory mediators involved and along with other inflammatory mediators implicated, it prevents the progression of periodontitis and periodontal tissue destruction. The unbalanced IL-6 levels could predict the early appearance of periodontitis more precisely than other periodontal pathogens in biofilms, and that serum IL-6 levels could be helpful in evaluating the degree extent of periodontitis [5].

It has been demonstrated that periodontal patients presented higher salivary IL-6 than healthy subjects and also a proportional increase of salivary IL-6 were associated with the extent of periodontitis and tooth loss [5].

Besides, it had been shown that patients with periodontitis presented significant higher serum and salivary galectin‐3 levels than nonperiodontal patients due to the fact that inflammation tissue fibrosis and angiogenesis are the main processes by which Gal-3 is involved [6].

Inflammatory periodontal diseases are treated primarily by supra- and subgingival debridement of affected tooth surfaces. Mechanical and surgical treatment combined with proper oral hygiene measures can arrest or prevent further periodontal attachment loss in most individuals. However, despite diligent dental therapy, some individuals continue to experience periodontal breakdown, may be due to the ability of major periodontal pathogens, like *Porphyromonas gingivalis* (PG), *Aggregatibacter actinomycetemcomitans* (AA), *Fusobacterium nucleatum* (FN), *Treponema denticola*, and bacteroides (TB), to invade periodontal tissues or to reside in furcations or other tooth structures outside the reach of periodontal instruments or due to poor host defense mechanisms [7].

Studies demonstrated that the use of systemic antimicrobial agents adjunctive to mechanical periodontal treatment may be an important therapeutic strategy in the treatment of periodontal diseases. [8] These results were observed with the use of different antibiotics, such as amoxicillin/metronidazole, azithromycin, clindamycin, and clarithromycin [8].

Throughout the years, different indications and motivations on prescribing antibiotics in addition to nonsurgical periodontal therapy have been presented: (a) diagnosis of aggressive periodontitis,(b) presence of deep periodontal pockets and disease severity, (c) bacterial invasion and activity of the disease, and (d) specific microbiological profiles of the subgingival plaque. Due to the absence of generally accepted guidelines, the decision of prescribing antibiotics with nonsurgical therapy is mainly subject to the personal experience of the clinician [9]. Nevertheless, nowadays, there is increasing global attention to the antibiotic exposure of the population due to development of resistance.

In light of this, a systematic literature review was carried out to assess the effectiveness of the addition of antibiotics after nonsurgical debridement of periodontal pocket during initial therapy and to compare different antimicrobial prescription protocols, proposed in the last five years, by assessing different associations, minimal effective dose, and optimal antimicrobial duration.

## 2. Materials and Methods

This systematic review was carried out and recorded according to Cochrane Handbook for Systematic Reviews of Interventions and Preferred Reporting Items for Systematic Reviews and Meta-Analyses (PRISMA).

In this systematic review, the **PICOT** format was used in formulating an evidence question, resulting in the following:

 
**P**: patients with periodontitis, nonsmokers with no associated systemic diseases or abnormalities, and patients not taking medications that may have a direct impact on periodontal treatment
 
**I**: intervention, this review aimed at studying the effectiveness of the adjunction of antibiotics during the initial phase of nonsurgical treatment
 
**C**: comparator, conventional nonsurgical periodontal therapy based on manual and/or ultrasonic scaling and root planning
 
**O**: outcome, improvement of clinical parameters in particular PD, CAL, and BOP during periodontal disease management
 
**T**: timing, laser application during the initial therapy in association with conventional mechanical instrumentation

### 2.1. Search Strategy

Two Internet sources were used to search appropriate papers satisfying the study purpose. These sources included the National Library of Medicine, Washington, DC (MEDLINE-PubMed), and EBSCOhost. Mendeley was used for managing bibliographies and citations.

For this comprehensive search, the two databases were searched for eligible studies from October 2015. A selection of “MeSH terms” was established to remove the high number of irrelevant papers in manual searches. The following search algorithm was used to explore databases, using Boolean operators (AND, OR): ((“Periodontal Pocket/drug therapy”[Mesh] OR “Periodontal Pocket/therapy”[Mesh]) OR (“Root Planing/methods”[Mesh]) OR “Dental Scaling/methods”[Mesh]) OR (“Periodontitis/drug therapy”[Mesh] OR “Periodontitis/therapy”[Mesh]) OR (“Periodontal Attachment Loss/drug therapy”[Mesh] OR “Periodontal Attachment Loss/therapy”[Mesh])) AND (“Anti-Bacterial Agents”[Mesh] OR antibiotics[Text Word]).

The search terms and strategies were similar in the process of exploring the other database (EBSCOhost).

### 2.2. Review Process

The two authors screened the titles and abstract and, in the end, selected studies for full-text review for potential eligibility. In case of a disagreement between reviewers, the decision about study eligibility was made by trying to reach a consensus between the two reviewers.

### 2.3. Screening and Selection

The study selection process was performed in two phases. In the first phase, the studies were analyzed according to the following inclusion criteria:

1. Randomized clinical trials
2. Papers written in the English or French languages
3. Studies conducted on humans, in good general health, diagnosed with periodontitis
4. Adequate information about the methodology, including the groups studied, sample size per group, and the study design for testing the hypothesis
5. Adequate information on the protocols followed
6. Adequate information on the outcome measures: clinical, microbiological, and/or biological parameters
7. Follow-up period that is more than 6 months

In the second phase, studies were excluded if they met one or more of the following exclusion criteria:

1. Patients with smoking habits, need for antibiotic premedication for routine dental therapy, antibiotic therapy in the previous 6 months, and allergy to MTZ, AMX, AZ, CLM, or chlorhexidine
2. Partial or incomplete data
3. Text of the article not available

### 2.4. Data Extraction

To conduct this systematic review, a personalized data extraction table was used for retrieving relevant data.

To avoid data extraction errors, the two reviewers made independent data collections and then confronted their results.

The data included authors' names, publication year, definition and diagnosis of periodontitis, participants' characteristics (age, gender), intervention undertaken (antibiotic/placebo regime), sample size, and length of follow-up.

### 2.5. Outcome Variables

The clinical parameters of PD reduction and CAL gain were the primary outcomes of interest.

Microbiological parameters were also assessed; they presented our secondary outcomes.

## 3. Results

**Figure 1:**
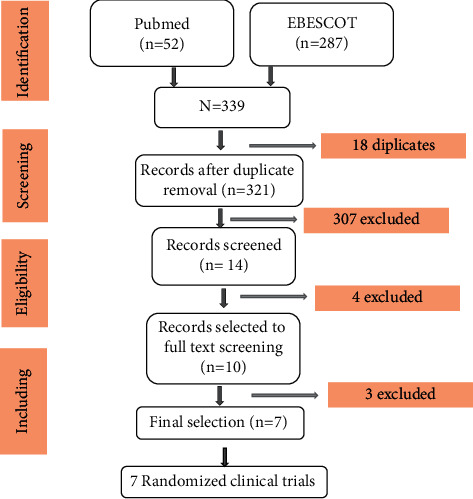
PRISMA flowchart of the study selection process.

The search results are presented in Figure 1. Only 7 articles made it and were therefore considered eligible for our review. All the included studies were RCTs. 100% of the included articles were studies with a high level of scientific evidence whose grade of recommendation is interesting.

### 3.1. Characteristics of the Included Studies

A summary of the included studies is given in Table 1. All the included studies were RCTs and were published between 2015 and 2020.

Clinical trials comprised 524 participants. Studies included a minimum of 30 [15] up to 180 [10] subjects.

The target population included generalized aggressive periodontitis (GAgP) in two studies [13, 16], chronic periodontitis (CP) in three studies [12, 14, 15], and moderate-to-severe periodontitis in two studies [10, 11]. The subgingival debridement regime ranged from a full‐mouth approach in one session [13, 16] to a staged approach over 14 days [11, 12, 14]. One study compared the full-mouth approach in two sessions within 24 hours with two sessions 21 days apart [10]. The full-mouth SRP procedure was not detailed in one study [15]. The antibiotic regimes varied too, including amoxicillin + metronidazole for one study [12], two cases comparing the amoxicillin + metronidazole with firstly metronidazole alone [14] and secondly with moxifloxacin [16], one study interested on metronidazole solely [10], one on azithromicin [11], and finally clarithromycin in two studies [13, 15]. A placebo was used for control patients in all included studies. The study follow‐up durations ranged from 6 to 12 months.

Clinical parameters were assessed in 5 studies [11, 13, 15, 16], and basically, PD and CAL were concerned. The microbiological parameters were searched in 6 articles [10, 11, 13–16]. Sites colonized by *Porphyromonas gingivalis*, archaea, *A. actinomycetemcomitans*, and/or *Tannerella forsythia* were assessed. The biological parameters were evaluated in one study [15] where C-reactive protein was measured.

### 3.2. Risk of Bias

There was no disagreement between the reviewers in assessing and evaluating the quality of the studies. All of the studies included in the systematic review showed a low risk of bias, with a score of 5 for all the included studies (Table 2).

## 4. Discussion

The results of the different studies showed that the addition of systemic antibiotics in the active phase of periodontal treatment favors improvement in clinical, microbiological, and/or biological parameters (attachment gain and PD reduction, even though it did not lead to a reduction in BOP when compared to the SRP-treated groups and the placebo). The seven RCTs included differed in terms of study population, sample size, risk of bias, statistical methods applied, primary outcome, administration of placebo, start of medication, ATB classes, dosage, and/or sequencing of nonsurgical treatment. These differences made comparison of different protocols not easy to do.

### 4.1. Clinical Parameters

Five of the seven studies (71.4%) [11, 13, 15, 16] included in our review assessed the clinical effects of the antibiotic adjunction on the treatment of periodontitis. Two of them [13, 16] are considered with general aggressive periodontal patients, one [11] with moderate-to-severe periodontitis, two [12, 15] with chronic severe periodontitis.

These five studies showed that the addition of antibiotics, whatsoever is the antimicrobial agent, provides additional clinical benefits: in all of them, the PD was significantly reduced compared to the placebo group. According to Andere [13], this statistically significant reduction was observed when PD ≥ 7 mm after 6 months of initial therapy. The same was proved by Carlos [16]; the only difference is that this outcome is obtained when PD ≥ 6 mm instead of 7 mm. According to Santosh, the mean reduction of PD from baseline is 2.82 mm and 1.31 mm after 6 months in the test and control group, respectively (*p* < 0.001). After 12 months, this reduction is about 2.91 mm and 1.51 mm in the test and control group, respectively.

The CAL was significantly improved in two studies (40%) [11, 12]. The CAL gain was significant only when PD ≥ 6 mm, according to Carlos [16]. In one RCT (20%) [10], the CAL was gained but not in a significant way, and in other one (20%) [13], no additional benefits were observed in terms of CAL gain (*p* = 0.3).

BOP was assessed in three included RCTs [11, 12, 16] (60%). In two studies [12, 16], it was significantly reduced and in one [11], it was just significant at 6 months only (*p* = 0.0278). This reduction is about 52.65% and 47.31% in the test and control group, respectively, after 6 months.

The four antibiotic groups mentioned in the study conducted by Ivan [12] had a greater reduction in the percentage of sites with BOP, in mean PD, and in gain of CAL between baseline and 1 year (*p* < .05). The lowest mean number of sites with PD ≥ 5 mm and ≥6 mm at 6 months and 1 year was observed in the two groups taking antibiotics for 14 days (*p* < 0.05). These two groups also had a higher mean reduction in sites with PD ≥ 5 mm from baseline to 1 year in comparison with the control group (*p* < .05). However, no statistically significant differences were observed between the two dosage subgroups (250 mg or 400 mg of metronidazole). Hence, we can conclude that the minimal effective dose of MET associated with AMX in this study is 250 mg. No addition benefits were obtained from increasing the dose which was not the case for the time of antibiotic intake; the two subgroups taking the antibiotics for either 7 or 14 days differed significantly for several parameters.

### 4.2. Microbiological Parameters

*A. actinomycetemcomitans*, a Gram-negative, facultatively anaerobic coccobacillus, is a bacterium with an array of diverse potential virulence characteristics, including multiple immune evasion mechanisms and novel mechanisms for binding to host matrices and invading host cells [17].

The *Aggregatibacter actinomycetemcomitans* (AA) reduction was evaluated in four studies [11, 13, 15, 16]. A significant reduction when comparing the test group to the control group was proved in all of them but in one [13]. This difference was detected until 12 months after the initial phase of therapy in the study conducted by Santosh [11] using Az as ATB (*p* < 0.001). According to Andere [13], when AA counts were analyzed, the CLM group showed a statistically significant higher continuous reduction at 6-month follow-up than at the 3 months which was not noticed in the placebo group, but when the two groups were compared, no statistically significant differences were observed. However, according to Suryaprasanna [15], with the adjunction of CLM, this improvement was significant until 3 months after initial therapy but not after 6 months. For Carlos [16], even after 6 months, the adjunction of the MOX or AMX + MET has a significant impact on AA reduction; however, adjunctive MOX diminished subgingival AA to unnoticeable levels. In addition, only patients taking AMOX + ME reported adverse events. Therefore, adjunctive MOX should be considered a relevant alternative in the treatment of general aggressive periodontitis.

A significant reduction in the level of PG was proved in the three studies [10, 12, 15] that assess this bacterium in subgingival samples after the adjunction of the ATB, compared to the placebo group. This reduction was observed significantly in the group using full-mouth disinfection (FMID) associated with MET after 3 and 12 months but not in the group associating SRP with MET according to Hans [10]. Also, in the study conducted by Andere [13], CLM showed statistically significant higher reduction of this species at 6 months (*p* = 0.0001) than in the placebo group; however, from 3 to 6 months of follow-up, there was a statistically significant decrease in PG for the CLM group compared to the placebo group. For Suryaprasanna [15], this significant difference between test and control groups was observed until the 3^rd^ month but not significant (*p* = 0.774) at 6 months after initial therapy.

According to Andere [13], adequate scaling and root planning and biofilm control is enough to promote PG reduction at 3 months. However, in the long term, it may be necessary to associate an antimicrobial to avoid the reestablishment of this microbial load [13].

Sites colonized by archaea and its levels were assessed in one study conducted by Ramiro [14]. At 6 months, both test groups (AMX + MET or MET only) had lower percentage of sites colonized by archaea and lower mean levels of this species at initially deep pockets (PD ≥ 5 mm) than in the control group [14]. No significant difference was observed between the two test groups. The author explained these results by the susceptibility of archaea to MTZ [14].

*Tannerella forsythia* (TF) was evaluated in one study [10], which was considered in moderate-to-severe periodontal patients and concluded that this species was significantly reduced after the adjunction of MET combined with FDIS at 3 and 12 months. Therefore, this combination FDIS + MET appeared statistically more effective in reducing both PG and TF below detection levels for up to 12 months following treatment compared to SRP + placebo, FDIS + placebo, and SRP + MET. This result might suggest that the FDIS does indeed achieve the purported reduction of the risk of reinfection of a previously disinfected area before the completion of the SRP treatment.

### 4.3. Biological Parameters

C-reactive protein (CRP) elevation is a part of the acute phase response to acute and chronic inflammation and particularly in periodontitis [18].

One study [15] assessed the CRP level and concluded that there is no significant reduction in the level of CRP between placebo and test groups.

Three studies [12, 14, 16] considered the association of amoxicillin/metronidazole. While amoxicillin dose (500 mg) was the same in the three studies, the metronidazole dose ranged from 250 mg to 500 mg. Significant improvement in the clinical and/or microbiological parameters was observed whatsoever the dose. Even Ivan Borges [12] demonstrated that there was no difference in the final outcome regardless of whether 250 or 400 mg of metronidazole dose was adopted. It was explained by the fact that the concentration of MTZ in the gingival crevicular fluid would probably plateau after a daily dose of 750 mg.

The duration of the administration ranged from 7 to 14 days. But, it has been proved that 14 days were more effective than 7 days. Ivan [12] explained it by the fact that longer periods of exposure to antibiotics would be required to kill microorganisms living in the highly organized subgingival biofilm structure. A longer exposure to amoxicillin/metronidazole would probably maintain low bacterial levels and allow more time for recolonization of the recent scaling [12].

Metronidazole alone was adopted in two studies [10,14], and it proved its effectiveness on archaea.

Andere [13] and Suryaprasanna [15] both used clarithromycin as an adjunctive to the SRP but they adopted different protocols; the former prescribed 500 mg of this molecule twice a day for 3 days, and the latter prescribed 500 mg thrice a day for 7 days. The first protocol obtained significant clinical improvement but only in sites with PD ≥ 7 mm, while the second showed that the PD reduction was significant in all sites without exceptions.

**Figure 2:**
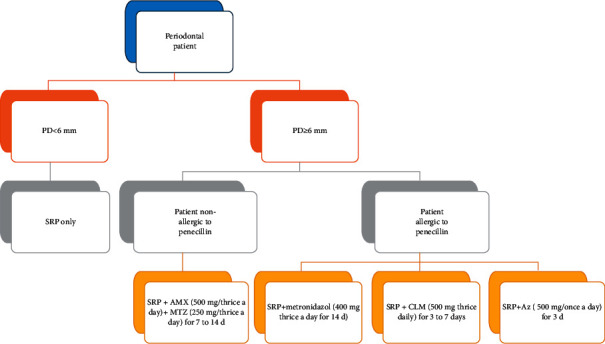
Summary of different effective suggested protocols.

According to EFP, the adjunctive use of specific systemic antibiotics may be considered for specific patient categories, e.g., generalized periodontitis stage III in young adults or refractory periodontitis [9]. Once it is the indication, different protocols were suggested and have proven their effectiveness (Figure 2).

## 5. Conclusion

From this systematic review, the following can be concluded:

  The overall findings of this review show an additional effect of the use of antibiotics as an adjunctive to NSPT.
  Sites with PD > 6 mm may benefit most from the adjunctive use of antibiotics in NSPT.
  The heterogeneity of the study subjects, prescription parameters, and SRP sequencing procedure makes protocols comparison not possible. However, the protocol based on prescription of amoxicillin + metronidazole seems to be more effective and it showed better results, but the dose and the duration of prescription are still controversial.
  The full-mouth approach in one or two sessions within 24 hours is recommended whenever possible to avoid the recontamination of sites already treated.

## Figures and Tables

**Table 1:** Characteristics of the included RCTs.

Author and year	Diagnosis	Population	Mean age	SR = *F*/M	PD/CAL	ATB, dose, duration		Description of the intervention					Outcome (comparing the test group with the control group)	Follow- up
Hans [10] 2015	Moderate-to-severe periodontitis	180	[35, 75]	-	≥5 mm	MTZ (400 mg/thrice a day) for 10 d		SRP + placebo	FDIS + placebo		SRP + MET	FDIS + MET	MP: significant reduction of PG and TF just in the FDIS + MET group after 3 and 12 months	12 months
Martande et al. [11] 2016	Moderate-to-severe periodontitis	70	[25, 45]	—	>6 mm	Az (500 mg/once a day) for 3 d		SRP		SRP + AZ			CP: significant reduction of CAL and PD in the AZ group (*p* < 0.001) MP: significant reduction of subject positive to AA in the AZ group (*p* < 0.0001)	12 months
Borges et al. [12] 2017	Severe ChP	110	≤30 years	62/47	≥4 mm	AMX (500 mg/thrice a day)+ MTZ (250 mg/thrice a day) 7 or 14 d	AMX (500 mg/thrice a day)+ MTZ (400 mg/thrice a day) 7 or 14 d	SRP + placebo	SRP + AMX + 250 mg of MTZ for 7 d	SRP + AMX + 250 mg of MTZ for 14 d	SRP + AMX + 400 mg of MTZ for 7 d	SRP + AMX + 400 mg of MTZ for14 d	CP: significant reduction of CP in groups AMX + MET for 14 days compared to those for 7 days	12 months
Bechara Andere et al. [13] 2017	GAgP	40	32.2	38/2	≥6 sites, PD ≥ 5 mm and ≥2 sites, PD ≥ 7 mm	CLM (500 mg twice daily) for 3 d		FMUD + placebo		FMUD + CLM			CP: significant reduction in PD in sites with PD ≥ 7 mm after 6 months MP: significant reduction of PG and AA after 6 months	6 months
Ramiro et al. [14] 2018	GChP	59	≤30 years	36/23	≥5 mm	MTZ (400 mg/thrice a day) for 14 d	AMX (500 mg/thrice a day)+ MTZ (400 mg/thrice a day) for 14 d	SRP		SRP + MET		SRP + AMX + MET	MP: significant reduction of sites colonized by archaea with lower level in pockets with PD < 6 mm	6 months
Suryaprasanna et al. [15] 2018	ChP	30	[30,50]		≥5 mm	CLM (500 mg thrice daily) for 7 d		SRP			SRP + CLM		CP and BP: reduction of GI, CAL, and CRP but not in a significant way MP: significant reduction of AA and PG after 3 months but not 6 months	6 months
Ardila et al. [16] 2020	GAgP	36	≤30 years	23/13	≥5 mm	MOX (400 mg/once a day) for 7 d	AMX (500 mg/thrice a day)+ MTZ (500 mg/thrice a day) for 7 d	SRP	SRP + MOX		SRP + AMX + MET		CP: significant reduction of PD and CAL for PD ≥ 6 mm at 6 months MP: AA reduction significantly at 6 months	6 months
		524												

CAL = clinical attachment level, PD = probing depth, GI = gingival index, CRP= C-reactive protein, CLM= clarithromycin, FMUD = full-mouth ultrasonic debridement, AMX = amoxicillin, MTZ = metronidazole, MOX = moxifloxacin, SRP = scaling and root planing, FDIS = full-mouth disinfection, PG= *Porphyromonas gingivalis*, TF = *Tannerella forsythia*, and AA = *Aggregatibacter actinomycetemcomitans.*

**Table 2:** Jadad scores scale [6].

Reference	Randomization	Blinding	Withdraw	Appropriate randomization	Appropriate blinding	Score
Hans [10]	1	1	1	1	1	5
Martande et al. [11]	1	1	1	1	1	5
Borges et al. [12]	1	1	1	1	1	5
Bechara et al. [13]	1	1	1	1	1	5
Ramiro et al. [14]	1	1	1	1	1	5
Suryaprasanna et al. [15]	1	1	1	1	1	5
Ardila et al. [16]	1	1	1	1	1	5

## Data Availability

All the necessary data are included within the manuscript.
